# Neutrino Induced Doppler Broadening

**DOI:** 10.6028/jres.105.011

**Published:** 2000-02-01

**Authors:** J. Jolie, N. Stritt

**Affiliations:** Institut de Physique, Université de Fribourg, Pérolles, CH-1700 Fribourg, Switzerland

**Keywords:** crystal spectrometer, gamma-ray spectroscopy, interatomic potentials, neutrino helicity, phonon creation

## Abstract

When a nucleus undergoes beta decay via the electron capture reaction, it emits an electron neutrino. The neutrino emission gives a small recoil to the atom, which can be experimentally observed as a Doppler broadening on subsequently emitted gamma rays. Using the two-axis flat-crystal spectrometer GAMS4 and the electron capture reaction in ^152^Eu, the motion of atoms having an excess kinetic energy of 3 eV in the solid state was studied. It is shown how the motion of the atom during the first hundreds of femtoseconds can be reconstructed. The relevance of this knowledge for a new neutrino helicity experiment is discussed.

## 1. Introduction

During the last decade ultra-high resolution gamma-ray spectroscopy with the two-axis flat crystal spectrometer GAMS4 [1,2], installed at the high flux beam reactor of the Institut Laue-Langevin (ILL), Grenoble, France, in a ILL/NIST collaboration, has allowed the observation of very small Doppler broadening of gamma-ray transitions [3]. The measured broadening is of the order of Δ*E/E* = 10^–4^ to 10^–6^ and is induced by preceding high-energy gamma-ray emissions following neutron capture. The resolving power of the GAMS4 spectrometer, which is of the order of 10^–6^ even allows the measurement of the Doppler broadening caused by the thermal motion of the atoms in a solid state target [4]. The observation of such small broadenings led to the development of the GRID (Gamma Ray Induced Doppler broadening) method [4], which is used either to determine short lifetimes of nuclear excited states, or to study the slowing-down process of low-energy recoiling atoms in the bulk of the target. The latter permits the extraction of information on the form of the interatomic potential [5,6].

Besides the (n, γ) reaction, atomic electron capture provides both the excitation and the extra kinetic energy leading to Doppler broadened energy profiles of subsequently emitted gamma rays. Electron capture is a beta-decay process in which a nucleus captures an atomic electron and emits a neutrino which, in first approximation, is the only particle to leave the atom. Because the neutrino has a well defined energy, it induces a well-defined recoil velocity in contrast to normal beta decay which leads to a continuous range of initial recoil energies. Note that pure electron capture occurs only if the mass difference between the initial and final atom does not exceed 1022 keV, because then the competing β^+^ decay is forbidden.

The initial motivation to study Neutrino Induced Doppler broadening (NID) was the measurement of the neutrino helicity as proposed 10 years ago by H.G. Börner et al. [7]. The basic idea of this proposal was to redo the classical experiment of Goldhaber, Grodzins and Sunyar [8] in which the helicity of the neutrino is transferred to a Doppler-shift dependent circular polarisation of 963 keV gamma rays. In contrast to the earlier experiment the Doppler shift would be measured directly by GAMS4 without the need to rely on nuclear resonance fluorescence. An additional motivation was that the NID experiments allow the study of the slowing down process at very low kinetic energies [9]. At these energies the recoiling atom is not able to definitively leave its lattice site but instead performs vibrations around its equilibrium position.

## 2. Neutrino Induced Recoil

Beta decay associated with electron capture takes place via the following nuclear reaction:

$${}^{A}X_{N}+\;e\;\to {\;}^{A}Y_{N+1}\;+v.$$ (1)

which (as discussed above) results in a well defined value for the initial recoil energy if the Q value is lower than 1.022 MeV. Because of this upper limit on the Q-value the recoil energies that can be obtained are only of the order of a few eV for heavy nuclei. The recoil velocity of the newly formed atom is given from the conservation of momentum (for zero-rest mass neutrinos and using nonrelativistic kinematics) by:

$$\frac{\upsilon_{r}}{c}=\frac{Q_{\text{ec}}-\;B_{e}}{M\;\left({}^{A}Y\right)c^{2}}.$$ (2)

The *Q*_ec_ value for the reaction is defined as:

$$Q_{\text{ec}}=\;\left[M\left({}^{A}X\right)\;-M\left({}^{A}Y\right)\right]c^{2}.$$ (3)

In Eqs. (2) and (3) *M*(*^A^*X)*c*^2^ and *M*(*^A^*Y)*c*^2^ denote the atomic rest masses in the initial and final states and *B*_e_ is the binding energy of the captured electron. After electron capture a hole is created in an inner electron shell and the filling of this hole leads to secondary effects such as x-ray emission or the emission of Auger electrons. In the former case the recoil energy equals:

$$\frac{\upsilon_{r}}{c}=\frac{E_{\gamma }}{M\left({}^{A}Y\right)c^{2}}.$$ (4)

with *E*_γ_ the photon energy. When an Auger electron with energy *E*_e_ is emitted one has:

$$\frac{\upsilon_{r}}{c}=\frac{\sqrt{E_{e}\left(E_{e}+2m_{e}c^{2}\right)}}{M\left({}^{A}Y\right)c^{2}}.$$ (5)

**Fig. 1:**
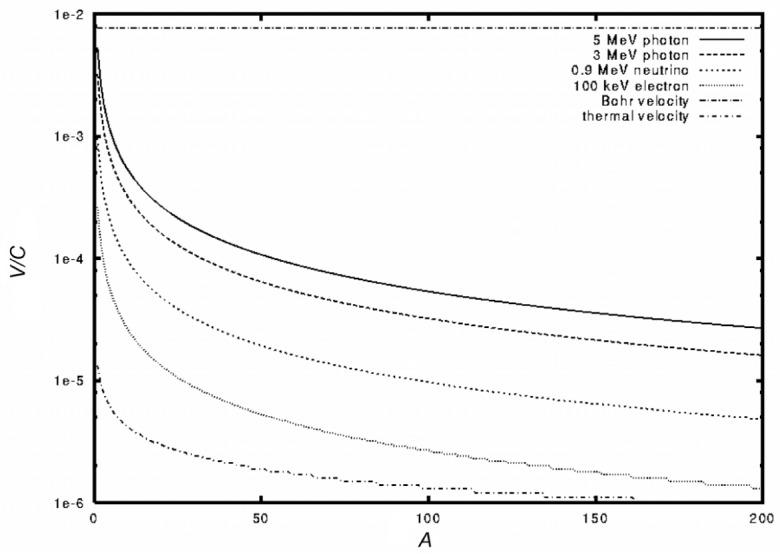
Comparison as a function *A* of the recoil velocities obtained after gamma-ray, neutrino and electron emission to the Bohr and thermal velocities.

Figure 1 compares the recoil velocities obtained by gamma-ray, neutrino or electron emission as a function of *A* with two other typical velocities: the thermal velocity υ_T_ associated with a maxwellian distribution:

$$D\left(\upsilon \right)=4\pi {\left(\frac{3}{2{\pi \upsilon }_{T}^{2}}\right)}^{3/2}\upsilon^{2}\;e^{-1.5(\frac{\upsilon }{\upsilon_{T}})2,}$$ (6)

and the typical Bohr velocity of an electron.

For NID the induced recoil is very low (3 eV in the Eu case) and so the recoiling atom is not able to definitively leave its equilibrium position. In order to analyse the measured line shapes different approaches can be followed. The first, *analytic*, approach is based on a phonon creation model, and was used to analyse the NID data in Ref. [9]. The second, *numerical*, approach relies on Molecular Dynamics (MD) simulations of the slowing-down process at ultra-low recoil energies, and is presented in [10–12]. Both approaches allow one to obtain information on either the lifetime of the nuclear state fed by the electron capture process, or to study the effect of ultra-low recoil energies on atoms located in a lattice of bulk matter.

## 3. Performed Experiments

All NID experiments have relied on electron capture in the isomeric 0^–^ state of ^152^Eu. In order to populate this state, which has a half life of 9.3 h, natural europium is used. Placed at the GAMS4 in-pile target position, the isotope ^151^Eu, which has a 48 % natural abundance, captures a thermal neutron and forms ^152^Eu. The cross section for this reaction is 9204 barns, making the targets black for thermal neutrons. Thus the neutron capture rate per cm^2^ of target area is limited to 2.5×10^14^ s^–1^. Following the electron capture a 1^–^ level at 963 keV in ^152^Sm is populated. This 1^–^ state decays to the nuclear ground state by emitting either a single 963.4 keV gamma ray, or a 841.4 keV to 121.8 keV gamma-ray cascade via the long-lived 2^+^ state. These three gamma rays were measured with the GAMS4 spectrometer. Two different kinds of targets have been used for the experiments. In the first measurements powder targets of different chemical composition were used [9]. These polycrystaline targets consisted out of Eu_2_O_3_, EuF_3_, EuF_2_ and EuCl_3_ powders. In the more recent experiments oriented single crystals of EuO were observed for different orientations towards the spectrometer [11,12]. EuO crystal has a fcc cubic structure and the NID measurements were performed once with the [100] direction towards the GAMS4 spectrometer and once with the [110] direction. Details on this experiment are given in ref [12]. Table 1 lists the thermal velocities υ_T_ (cf. Eq. (6)) which were measured using the 121.8 keV transition. This transition decays from a long-lived state such that the broadening is solely due to the thermal motion.

## 4. Description of the Slowing Down Process and Results

Before discussing the two different ways that were used to analyse the neutrino induced Doppler broadening line shapes, we note the main differences to those observed using gamma-ray induced Doppler broadening. The recoils induced in medium-heavy nuclei by electron capture are too small to create significant dammage of the regular lattice. Instead, after the initial recoil, the atom moves a small distance away from its equilibrium position, due to the extra kinetic energy. Because of this, it pulls the neighbouring atoms away from their positions, loses energy and slows down. When the recoiling atom has lost all of its kinetic energy, because of the forces exerted by the other atoms, it stops and starts to move back to its initial position. Now, regaining energy, the velocity will increase and reach a maximum near the equilibrium position. This time the velocity will be smaller than the initial one, since energy is dissipated in the crystal by pulling more and more atoms away from their equilibrium positions. Thus a lattice vibration, or phonon, is created. Since, as the name suggests, this is a collective process, it is sensible to test collective descriptions of the slowing down process. In contrast, in standard GRID measurements the recoil energies are about 400 eV and the velocity function of the recoiling atom is, to a good approximation, deduced from binary collisions until the atom moves at velocities in the order of the thermal motion of the target atoms [4]. In this respect the slowing down process can be described by considering individual two-body collisions. Another difference is the influence of the thermal motion itself which is important even for short lifetimes, because it is in the same order of magnitude as the recoil velocity.

To understand this process all data have been analyzed using two different approaches: the analytical phonon creation model and the semi-microscopic Molecular Dynamics simulations.

### 4.1 Analytic Description of the Slowing Down Process

For the slowing down of atoms with very low kinetic energies in solids the Phonon Creation Model (PCM) gives an analytic and simple description of the velocity. Assuming the Debye approximation and neglecting phonon-phonon interactions and incoherent thermal oscillations of the atoms in the lattice, the velocity of the recoiling atom for a isotropic medium or a monoatomic cubic Bravais lattice, is given by [13]:

$$\upsilon \left(t,\omega_{D}\right)=3\upsilon_{0}[\frac{2}{\omega_{D}^{2}t^{2}}\cos\left(\omega_{D}t\right)+(\frac{1}{\omega_{D}t}-\frac{2}{\omega_{D}^{3}t^{3}})\sin(\omega_{D}t)].$$ (7)

Equation [7] gives the velocity of the recoiling atom as a function of the time *t* and the Debye frequency ω_D_. In the Debye approximation the direction of the recoil is ignored because of the assumed isotropy and the neglection of temperature.

Employing the Debye approximation, the Doppler broadened line shape is described by:

$$I\left(E,\tau \right)=\int_{0}^{+\infty }I_{D}(E,\upsilon (t,\omega_{D}))\frac{N_{0}}{\tau }e^{\frac{-t}{\tau }}dt,$$ (8)

with *v*(*t*, ω_D_) given by Eq. (7) and *τ* the lifetime of the deexciting nuclear state. When neglecting the natural linewidth of the deexciting state *I*_D_(*E, v*) is approxi mately given by:

$$I_{D}(E,\upsilon )=\frac{c}{E_{0}\upsilon }\;\text{for}\;E_{0}(1-\frac{\upsilon }{c})<E<E_{0}(1+\frac{\upsilon }{c})$$ (9)

The integration of Eq. (8) is done numerically.

**Fig. 2:**
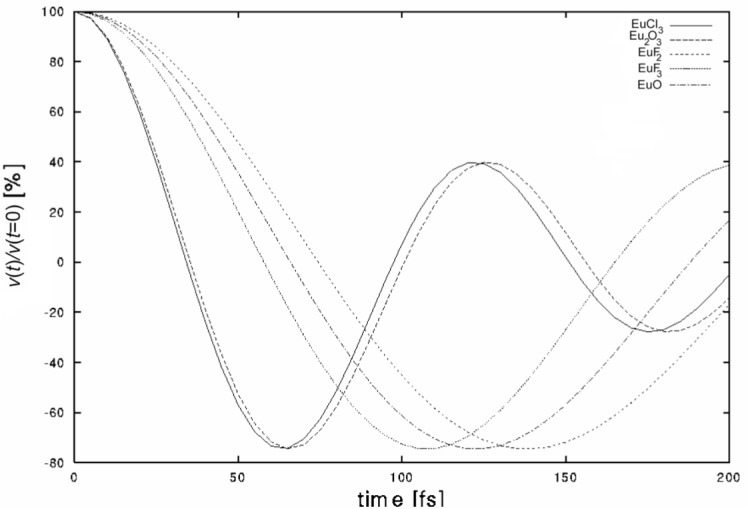
Time dependence of the recoil velocity obtained with the Phonon Creation Model for the different targets.

Because the initial recoil velocity, υ_r_/*c* = 6.54×10^–6^, is very close to the thermal velocity, one has to take account of the velocity spread due to the thermal motion. This is done by folding the theoretical line shape with a thermal width as explained in detail in Ref. [9]. Table 2 lists the deduced frequencies ω_D_ for all five targets using the lifetime value of τ= 29 fs obtained by Jungclaus et al. [14]. Note that all crystal effects connected to the EuO target were neglected. In this analysis also the effects of x-ray and Auger electron emission are neglected. Figure 2 shows the corresponding time-dependent velocities. One clearly notices the quick slowing down in Eu_2_O_3_ and EuCl_3_ compared with the other targets. For a helicity experiment it is clear that the ideal target would be EuF_2_. We have also tried to find an effect of a finite lifetime of the created phonons, but the fit of an exponentially damped Eq. (7) converged always to an infinite lifetime for the phonons. This shows that this effect is negligible when dealing with a nuclear lifetime of a few tens of fs while typical phonon lifetimes are about 1 ps.

### 4.2 Molecular Dynamics Description of the Slowing Down Process

Using Molecular Dynamics (MD) simulations and Monte Carlo simulations the description of the slowing down can be done in a much more detailed way [10]. By solving the Newtonian equations for atoms recoiling in random directions it is possible to study the spread in velocities and to treat in detail the effect of the thermal motion, x-ray and Auger-electron emission on the slowing down. For the MD simulations the main input is the interatomic potential which is treated as a set of pair potentials dependent on the distance *r_ij_* in between the atom *i* having charge *q_i_* and the atom *j* having charge *q_j_*. In the case of NID energies it was found that a Buckingham-type of potential describes best the data [10–12]. This potential has the form:

$$V\left(r_{ij}\right)=q_{i}q_{j}\frac{e^{2}}{4\pi \epsilon_{0}}\;\frac{1}{r_{ij}}\;+A_{ij}\;\exp\frac{-r_{ij}}{\rho_{ij}}-\frac{C_{ij}}{r_{ij}^{6}}$$ (10)

**Fig. 3:**
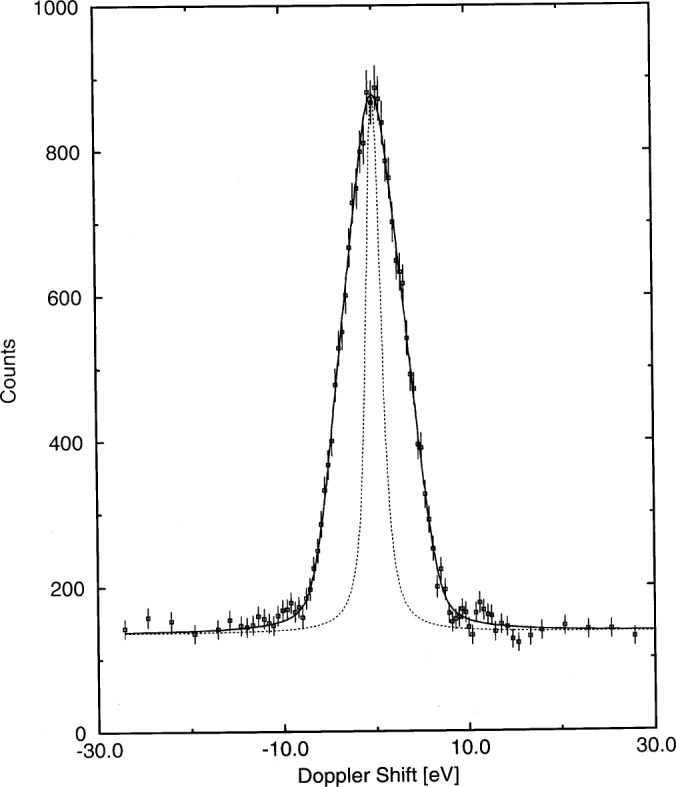
Comparison between fitted and measured line shape for the 841.6 keV transition in ^152^Sm. The dashed line gives the instrumental response [11].

Table 3 lists the parameters used for the analysis of the data. Knowing the interatomic potential the lifetime can be fitted and compared to those available in the literature. The experimental line shapes were analysed using the computer code GRIDDLE [15], and Fig. 3 illustrates the very good quality of the fit of the line shape for the EuO measurement [11]. The fitted lifetimes are given in Table 4 and a good agreement is obtained in comparison with the (n,n’γ) experiment, justifying the use of *τ*= 29 fs in the phonon creation model. In the study using the oriented EuO crystals a small, but observable, dependence of the Doppler broadening on the crystal orientation was found [12]. In contrast to the analytic description the MD simulations treat the thermal velocity and the emission of x rays and Auger electrons exactly.

A particular but albeit marginal problem encountered in the simulations, is Coulomb implosion of the simulation cell. In rare cases the atom reaches a very high charge state after an Auger cascade which leads by the attractive Coulomb force to the destruction of the lattice. Whether such an effect really takes place in nature is, however, not clear.

## 5. Discussion

From the MD simulations discussed above a detailed description of the slowing down is obtained which we analyse here in the context of the helicity measurement. Although EuF_2_ is the ideal target we will rely on the EuO data for our study, because the experimental data set was the best defined for EuO due to the use of oriented single crystals and the good statistics. The slowing down in EuO and EuF_2_ is also similar as was illustrated in Fig. 2.

**Fig. 4:**
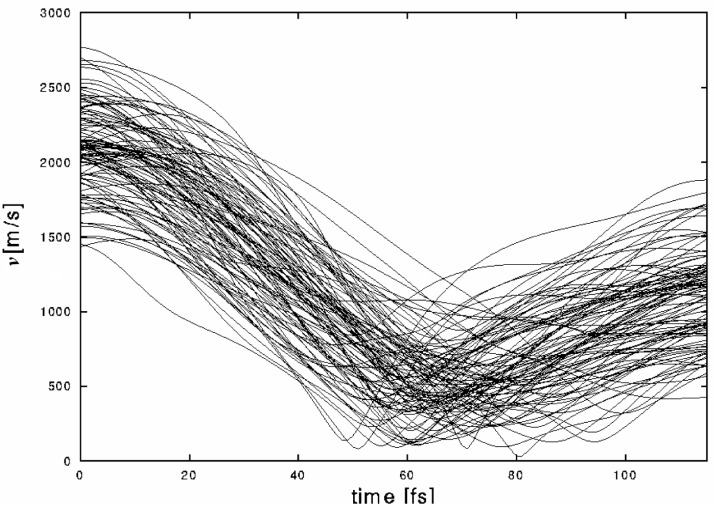
Individual recoil velocities of Eu recoiling in EuO as a function of time.

**Fig. 5:**
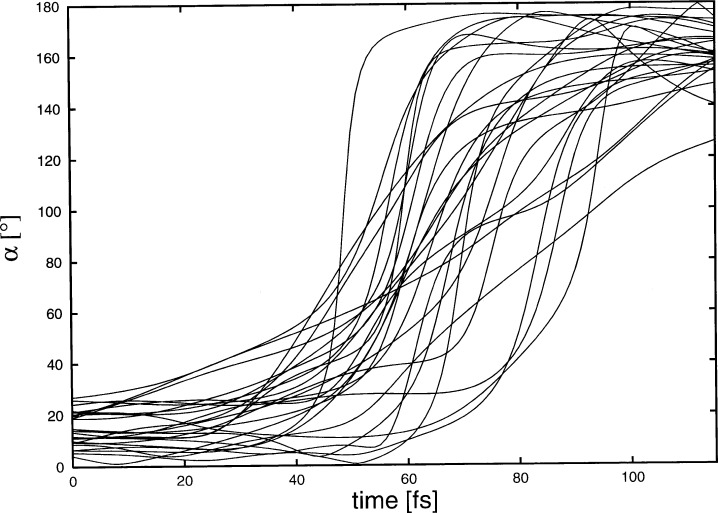
Time dependence of for *α* 25 individual recoils in EuO.

Before proceeding we recall three positive points: the dependence on crystal structure is small, the Auger cascades are rare and the lifetime of the 963 keV level in 152Sm is very short. However, a major problem remains. Figure 4 shows the velocities obtained for 100 individual trajectories in EuO. While the oscillatory character of this motion is clear by the turning point at 70 fs one notices a large velocity spread at any time. This spread is due to the thermal motion at the moment of the initial recoil. Because of this large effect we have studied the time dependence of the angle *α* (*t*), defined as the angle between the recoil velocity *υ* (*t*) at time *t* and the direction opposite to the neutrino momentum. This angle is directly related to the measurement of the neutrino helicity. This because the helicity:

$$H=\frac{p_{v}\cdot S_{v}}{\left|p_{v}\right|\cdot \left|S_{v}\right|}$$ (11)

is measured via the Doppler shift related to ***υ***(*t*) and any deviation from the ***p****_v_* direction is given by *α* (*t*). Figure 5 shows the values for *α*(*t*) for 25 individual recoils. At time *t* = 0 the averaged value for $\bar{\alpha \left(0\right)}$ equals already 12.5°. The behavior of this value as a function of temperature can be approximated by:

$$\bar{\alpha \left(0\right)}=\arctan\;\left(\frac{\upsilon_{T}}{\upsilon_{r}}\right),$$ (12)

yielding in our case 14°. The individual thermal velocities follow the broad Maxwell distribution and *υ*_T_ is only the root mean square velocity of this distribution, therefor individual atoms can have very low or high thermal velocities. This can be observed in Fig. 5. In some cases one finds atoms that perform either linear oscillations while other perform quasi-circular orbits around their equilibrium positions. In order to reduce this disturbing effect, which obscures the helicity measurement, the temperature should be reduced drastically. A spread of $\bar{\alpha \left(0\right)}$ of 1° corresponds for instance to a *υ*_T_ of 34 m/s occuring at 7 °K. This is clearly not attainable at an in-pile source.

At this stage one might wonder how Goldhaber et al. [8] were able to reach definite conclusions in their work. Besides the fact that they had only to show an effect, it should be remembered that they relied on difference measurements and varied the composition of the target. The slowing down only affects the information on the neutrino momentum independent from the one on the neutrino spin. The measurement of the difference in circular polarisation allowed than the elimination of gross effects connected to the slowing down or temperature. Nevertheless, for a very precise measurement these effect become dominant.

## 6. Conclusions

Over the last years we have analysed the different Neutrino Induced Doppler broadening experiments using Eu compounds as targets. Except for the problems associated with Auger cascades, a good understanding of the slowing down process at kinetic energies around 3 eV was obtained with the Molecular Dynamics simulations. Moreover, the lifetime of the 963 keV state in ^152^Sm could be determined to be (28.7±1.0) fs. This knowledge was then used to study the rôle of the slowing down and the temperature on the measurement of the neutrino helicity. We found important effects which need to be considered in detail if one wants to obtain a precise measurement of the helicity. We consider that now all data needed for a full Monte Carlo simulation of the experiment proposed in [7] are available.

## Figures and Tables

**Table 1:** Fitted values of the thermal velocity *υ*_T_

	Eu_2_O_3_, EuF_3_	EuF_2_, EuCl_3_	EuO
*υ*_T_ [m/s]	515 (51)	414 (57)	537 (24)

**Table 2:** Adopted values for *ω*_D_ for the different target compositions using the procedure described in the text and *τ* = 29 fs.

	Eu_2_O_3_	EuF_3_	EuCl_3_	EuF_2_	EuO
*ω*_D_[10^13^ s^–1^]	5.9 ± 1.06	3.6 ± 0.7	6.1 ± 1.0	2.8 ± 0.4	3.16 ± 0.15

**Table 3:** Values of the parameters for the Buckingham pair potential

*i–j*	*A_ij_* (eV)	*ρ_ij_* (Å)	*C_ij_* (eV Å^6^)	*q_j_* (e)
Eu – Eu	1715	0.317	0.0	+2,+3
Eu – F	3429	0.280	14.0	–1
F – F	369	0.280	12.5	–1
Eu – Cl	3886	0.349	169.6	–1
Cl – Cl	7911	0.383	2027.0	–1
Eu – O	5045	0.290	34.0	–2
O – O	22764	0.149	27.9	–2

**Table 4:** Lifetime values for the 963 keV level. Columns 1–5 are obtained from [10] and column 6 from [12]. They are compared to the lifetimes obtained using the (n,n′γ) reaction [14], nuclear resonance fluorescence [16] and the GRID technique [17].

	Eu_2_O_3_	EuF_3_	EuCl_3_	EuF_2_	average	EuO	(n,n′γ)	(γ,γ′)	GRID
τ [fs]	27.8 ± 2.8	22.4 ± 2.9	36.8 ± 2.8	24.2 ± 2.7	28 ± 6	28.7 ± 1.0	29 ± 4	41 ± 3	28 ± 12
